# American High School Students’ Knowledge and Beliefs about Parenting and Early Childhood Development

**DOI:** 10.3390/children10010025

**Published:** 2022-12-23

**Authors:** Eleanor O’Donnell Weber, Joseph Clark McIntyre, Meredith L. Rowe

**Affiliations:** 1 Independent Researcher, Charlottesville, VA 22901, USA; 2 Graduate School of Education, Harvard University, 6 Appian Way, Cambridge, MA 02138, USA

**Keywords:** parent education, child development, high school students, adolescent attitudes, surveys

## Abstract

Income-based achievement gaps in cognitive skills are already large when children enter Kindergarten. By adopting a preventative approach that considers the efficacy of providing parenting knowledge to individuals before they become parents while they are still in secondary school, we may be able to reduce achievement gaps. In this study, we examined adolescents’ knowledge and understanding of parenting and child development by creating and validating the Adolescent Parenting Knowledge and Attitudes Survey and administering it to over 1000 US high school students. This study shows that while many high school students hold beliefs consistent with successful outcomes for young children and their learning, there is much room for increasing their knowledge. The findings are discussed as presenting a potential opportunity to use high school as a site to improve adolescents’ knowledge and attitudes related to child rearing and development.

## 1. Introduction

How do people come to know how to be a parent? Are attitudes and knowledge about parenting and child development instinctual, learned, or simply the product of the experiences one has had with a child or with children? One theory posits that beliefs about child rearing and child development are developed through three means:

1. Beliefs come directly (and unquestioned) through the culture.
2. Beliefs are formed through the holder’s own childhood, family, and parenting experiences.
3. Beliefs are influenced by the exchange of ideas and assumptions of people from different cultures [1].

However, few studies have examined exactly how these three sources of beliefs and knowledge interplay in the development of parenting beliefs and the relative effects they have on what people come to believe about how children should be raised. This study attempts to contribute to that gap by examining the beliefs and knowledge of adolescents about parenting and child development, and the influences that have shaped those beliefs. 

High school students, most of whom are not parents yet and will not be for many years (if ever), are an especially interesting population to study for three reasons. First, late adolescence is nearing the end of the intensive period of being parented. Late teens are typically still living in the family home with their parents before leaving for college or establishing their own home. Therefore, they are in a unique position where they may have the developmental capacity to reflect upon their own childhood, family, and parenting experiences, while still being embedded within those experiences. Second, while still in this intensive period of being parented, they are at a developmental stage where they may be critically reflective about their relationship with their own parents and begin to think about what they want for their own futures [2] and future families. Finally, they are nearing the end of the compulsory education cycle where they are expected to have gained competencies for adulthood, and some may have even learned about child development and/or parenting in a formal classroom setting. In fact, more than half of all US states have at least one high school standard relating to parenting and child development, indicating that there is some recognition by education officials of the importance of parenting knowledge among adolescents for a healthy society. Yet, anecdotal evidence suggests that most states are not very active in attempting to meet those standards, nor is progress on meeting those standards assessed in any systematic way. This evidence is supported by the findings of this study as well. 

Understanding beliefs about parenting and child development is important since there is evidence that parenting styles differ across cultures and social classes [3], and that even within cultural and socio-economic groups, some parenting practices lead to better developmental outcomes for children [4]. Furthermore, parents who know more about child development and early learning tend to raise children who are better prepared for academic success in kindergarten [5,6]. This is particularly important since there is a well-documented and pernicious achievement gap by socio-economic class in the US, apparent as early as three years of age [7,8,9,10]. Moreover, children who start behind are more likely to stay behind relative to their more affluent and higher achieving peers [11], even despite such efforts as public pre-K [12]. Thus, it is vital to better understand what adolescents, most of whom will likely be parents in the future or may otherwise be able to influence childrearing within their circles of influence, know about parenting and child development. This study provides a preliminary understanding of what a broad sample of American high school students believes and has learned—through observation, direct experience caring for young children, school curricula, and other means—about parenting and child development and how those sources of information contribute to their knowledge and beliefs. Our research questions are as follows:

1. What does a diverse population of American high school students know and believe about several aspects of parenting and child development, particularly relating to those aspects of parenting and child development that are believed to promote early learning?
2. Do students with different characteristics (e.g., cultural, geographic, or socio-economic) or students who have had different experiences (who have more experience caring for young children, who have taken a babysitting class, or who are from a state with standards relating to parenting skills or child development) hold different attitudes and knowledge about parenting and child development?
3. What is the relationship between knowledge of child development, demographic characteristics, childcare experience, and beliefs about parenting and child development?

To answer the third research question, we tested a conceptual model of knowledge of child development as mediating personal characteristics and experiences with beliefs about parenting and child development.

## 2. Materials and Methods

### 2.1. Participants and Procedure

We developed and validated the Adolescent Parenting Knowledge and Attitudes Survey (APKAS) for use in this study. The APKAS was administered to 1044 American high school students recruited by Qualtrics, a leading survey research company, and completed online on the Qualtrics platform (survey data available upon request from the corresponding author) in July and August of 2018. Respondents were recruited via Qualtrics: Adult Qualtrics panel members—individuals who had joined Qualtrics to complete surveys on a variety of topics in exchange for small incentives such as cash, magazine subscriptions, or travel points—who reported having a high school-aged child in the home were invited to participate. If the parent/guardian agreed to allow the high school-aged child to participate, if the high school student himself/herself also agreed to participate, and if they met demographic criteria (see below about demographic quotas), they were able to access the full survey. The questionnaire took, on average, approximately fifteen minutes to complete and was able to be completed via computer or mobile device. To be eligible for the survey, respondents indicated that they are enrolled in high school (grades 9–12) in the United States. They also had to assent to taking the survey, and (if they were minors) were instructed to seek parental permission to take the survey (per Harvard University’s Institutional Review Board’s requirement). While partially completed surveys were collected and saved, all analyses took place using only the results of fully completed surveys.

We attempted to recruit a sample that reflects US demographic trends by setting quotas for certain groups using Qualtrics while also balancing the research priorities for this study (such as prioritizing older students and students from lower-income backgrounds, since older students and lower-income students may be the eventual targets of interventions to teach parenting and child development in high schools). We used data from the US Census Bureau’s Current Population Survey (October 2016) to approximate the number of adolescents to recruit from each demographic category. Qualtrics’ recruitment system allowed us to specify the number of respondents in a variety of categories, including sex, age, racial/ethnic background, region, and family income level. In Table 1, we describe the final quotas set for the sample.

### 2.2. Measures

The APKAS consists of 78 items to understand what high school students know and believe about the role of parents in childrearing, especially related to early learning, and what they know and believe about the child development process. The 78 questions can be broken down into the following 7 substantive categories and 3 categories about background and experience. For items about beliefs (categories 1-6 below), respondents could answer “(5) Strongly Agree”, “(4) Agree”, “(3) Neutral”, “(2) Disagree”, “(1) Strongly Disagree”, or “(0) Don’t know”. For items about their knowledge of child development (KOFCD), respondents could answer “True”, “False”, or “Don’t know”.:

1. Active Learning: Belief about the importance of active learning opportunities for children [6 items, Cronbach’s α = 0.74]: These questions are about whether the respondent believes that children’s learning is an active process, where children should have the opportunity to have freedom and be able to try things themselves.
2. Empathy: Belief about the importance of empathetic awareness and social-emotional learning [7 items, Cronbach’s α = 0.74]: These items aim to understand the extent to which the respondent believes that children’s emotional needs are important and that it is important for parents to promote their child’s socio-emotional development.
3. Growth: Belief about the importance of holding and promoting a growth mindset [5 items, Cronbach’s α = 0.72]: This series of questions aims to uncover the extent to which the respondent believes that intelligence is innate versus can be nurtured, especially by parents.
4. Oral Language Development: Belief about the importance of supporting oral language development [6 items, Cronbach’s α = 0.78]: These questions are related to the respondents’ beliefs about how children acquire oral language and the role that parents and caregivers can play in this process.
5. Parenting: Belief about the importance of parenting knowledge and efficacy [6 items, Cronbach’s α = 0.71]: These questions relate to the extent to which participants believe that parenting requires certain skills and dispositions and whether these can be taught.
6. Role: Belief about the importance of the role parents can play in early learning including in literacy and math [5 items, Cronbach’s α = 0.69]: As with the category above, these questions seek to understand whether the respondents believe that parents and caregivers should play an active role in children’s early learning, including in developing literacy and math skills.
7. KOFCD: Knowledge of child development index [7 items, Cronbach’s α = 0.31]: Most of the other categories relate to the respondents’ beliefs, whereas this category is meant to explore whether the respondents know about typical patterns of child development in the United States. Respondents may overestimate or underestimate (or correctly answer) questions about what children can are capable of at different ages.

The survey also measures the following information:

1. Respondents’ biographic information [26 items]: Including gender, age, grade, academic achievement, parents’ educational attainment, childcare experience, and school type.
2. Sources that inform the respondents’ knowledge and attitudes [5 items]: We were interested in where high schoolers believe they have learned about parenting and child development, and where they think they will go for information in the future. Adolescents likely have not been exposed to parenting “experts” (except in the ways that their parents have used expert advice in order to raise them), and do not have their own parenting experience to draw upon.
3. Final questions [5 items]: The few final questions asked whether we missed anything (open), whether the questionnaire made the respondent think differently (open), whether the respondent feels ready to be a parent yet (y/n), the most important thing a parent should do (open), and what the hardest part of being a parent is (open).

For more information about the development of the APKAS instrument and its validity, please see the supplementary information to this article.

### 2.3. Data Analysis

We first analyzed the results of the questionnaire to provide a picture of what American high school students know and believe about parenting and child development through descriptive statistics. Next, we analyzed the data by various subgroups (geographic (states with standards versus without; by respondent-reported zip code (using data from the US Census to determine urban versus rural and mean household income)), ethnicity, gender, experience caring for young children) using *t*-tests, linear regression, and structural equation modeling (SEM) to understand how parenting and child development knowledge and attitudes differ across subgroups. For regression and SEM analysis, “Don’t know” answers were excluded using a full information maximum likelihood estimator to account for the missingness. 

## 3. Results

First, our goal is to understand adolescents’ beliefs and knowledge about parenting and child development. Overall, between two-thirds and four-fifths of high school students in the sample hold beliefs that are associated with healthy development and cognitive benefits for young children: Across most questions, 60–80% of respondents answered with either “Agree” or “Strongly Agree” (see Table 2). However, for most items, fewer than 50% “Strongly Agree” (the “preferred” response for most items) with statements consistent with beliefs associated with positive outcomes for young children. Participants scored much lower on items that assessed their knowledge of child development and developmental milestones. In the sections that follow, we present the participants’ responses by domain, including active learning, empathetic awareness, socio-emotional learning, growth mindset, oral language development, parenting knowledge and efficacy, role in early learning, and knowledge of child development. We also report the key sources that adolescents report they draw upon to inform their knowledge of parenting and child development, as well as other factors including the gender, socio-economic context, and prior childcare experience of the respondent. Finally, we tested our conceptual model of knowledge of child development as mediating personal characteristics and experiences with beliefs about parenting and child development.

All of the belief categories were correlated with one another. We created scale scores for each section of the APKAS by converting responses into numbers and averaging across all the items in a section (after first reverse-coding negatively balanced items). Using these scale scores, we found that each of the belief categories was at least moderately correlated with one another, with the weakest correlations between the importance of the role of parents in learning and empathy and socio-emotional learning and the strongest between beliefs about the importance of oral language development and empathy and socio-emotional learning (See Table 3). 

### 3.1. Belief Domains

#### 3.1.1. Active Learning

Participants appear to believe that parents should promote active learning practices in their children. In particular, most agree strongly that children should have opportunities to learn about things they are interested in (54% “Strongly Agree” and 33% “Agree”). Respondents also seem to believe that children learn well when they have a chance to try things for themselves (38% “Strongly Agree” and 45% “Agree”) and that parents should encourage their children to be curious, explore, and question things (30% “Strongly Agree” and 44% “Agree”). However, one-quarter of high school students in the sample feel neutral about whether parents should teach children to do things for themselves, and almost 50% either believe that a three-year-old is too young to help make his or her own sandwich or feel neutral about it.

#### 3.1.2. Empathetic Awareness and Socio-Emotional Learning

For the most part, participants in the study believe that empathy is an important learned skill and socio-emotional learning is important for young children. Furthermore, 56% of participants Strongly Agree” and 31% “Agree” that learning to play nicely with others is very important for young children, and slightly fewer (48% “Strongly Agree” and 37% “Agree”) believe that it is important for children to learn to recognize feelings in others. On the other hand, participants may have been less sure about the importance of parents being empathetic toward their children: 41% of high school students in the sample believe that parents who are sensitive to their children’s feelings and moods often spoil them (and 7% “Don’t know” and 31% feel “Neutral”).

#### 3.1.3. Growth Mindset

In general, high school students in the sample seem to believe that holding a growth mindset—including strong beliefs that praising hard work (47% “Strongly Agree and 35% “Agree”) and encouraging children to try their best (61% “Strongly Agree” and 27% “Agree”)—is important for both parents and children. Similarly, high school students in the sample also believe that parents can play a big role in children’s learning, no matter how smart the child is (51% “Strongly Agree” and 33% “Agree”) and believe that people can learn to be better parents (48% “Strongly Agree” and 36% “Agree”). While the majority of students seemed to hold beliefs about the importance of a growth mindset, around 10% of respondents felt neutral about whether a growth mindset is important. 

#### 3.1.4. Oral Language Development

Participants indicated that they have strong beliefs regarding the importance of parents speaking to infants (48% “Strongly Agree” and 35% “Agree”), giving children opportunities to speak as well as to listen (46% “Strongly Agree” and 40% “Agree”), and reading to children (38% “Strongly Agree” and 41% “Agree”). However, when asked about items related to how to develop oral language (such as by using full explanations when possible, using word games, or using all kinds of words), participants seemed less sure. For example, when asked to respond to, “Playing a game like “20 questions” is not only fun but can also help young children learn new words”, respondents felt less strongly: Only 24% “Strongly Agree” and 41% “Agree”, and 23% felt neutral. Similarly, when asked about using both simple and complex words when talking to young children, 26% “Strongly Agree”, 37% “Agree”, 22% felt neutral, 8% “Disagree”, and 2% “Strongly Disagree”—the second highest rates of disagreement for any positively worded item. Finally, in response to the item, “When a child asks a question, the parent should give a full explanation”, 22% “Strongly Agree”, 37% “Agree”, and 35% felt neutral.

#### 3.1.5. Parenting Knowledge and Efficacy

Approximately two-thirds of participants in this study believe that parenting is hard, it helps to know a lot about children to be a good parent, and that they are capable of being good parents someday. However, 6% “Disagreed” and 2% “Strongly Disagreed” that it helps to know a lot to be a good parent. Participants feel more strongly that parents should control their temper (47% “Strongly Agree” and 35% “Agree”) and that parents should be patient (56% “Strongly Agree” and 32% “Agree”). Only 16% “Strongly Agree” and 28% “Agree” that parents who nurture themselves make better parents, 35% feel neutral, 14% report that they “Don’t know”, 5% “Disagree”, and 1.5% “Strongly Disagree”. 

#### 3.1.6. Role in Early Learning

While, in general, participants indicated that they have strong positive beliefs regarding the importance of the parent’s role in promoting early learning, 10–37% feel neutral about what role parents should play. For example, 41% of respondents “Strongly Agree” and 42% “Agree” that it is good for children’s development if parents play with them, but 10% feel neutral and 4% either “Disagree” or “Strongly Disagree”. Furthermore, 29% of participants feel neutral about whether it is the parent’s job to teach the alphabet and how to count to ten before school starts and 37% feel neutral about whether parents should have high expectations for their children. In fact, 14% “Disagree” and 4% “Strongly Disagree” that parents should have high expectations—the highest rate of disagreement for a positively worded item on the APKAS. 

### 3.2. Influences on Beliefs

#### 3.2.1. Knowledge of Child Development

We included a brief index of items that focused on adolescents’ knowledge of what children are capable of at different ages and how they learn and communicate. Overall, it seems that high school students have limited knowledge of many of the commonly agreed-upon developmental milestones. They are unsure of what infants and toddlers are capable of at different ages and seem not to ascribe much agency to young children. For example, a large proportion of respondents “Don’t Know” whether babies as young as 2 months old can feel bored (34%) and whether children typically say their first word at approximately 6 months old (44%). On the other hand, approximately 75% of high school students believe that toddlers’ pointing is meaningful, which is heartening (see Table 4). When asked to respond to, “Children age four and under are too young to do many things for themselves”, 51% of high school students agree. 

Fewer than half of respondents answered with the correct response on four out of six of the knowledge questions, which is particularly interesting since the participants had a fifty–fifty chance of choosing that answer (assuming they did not pick “Don’t know) if they simply guessed. A large number do not believe four-year-old children are capable of doing many things (51%, as noted above) and do not believe young infants can feel bored (21%). Some respondents overestimated what children should be able to do: 14% believe that one-year-old children know right from wrong, and 42% believe that one-year-old children are able to stay away from things that can harm them. There is not a single item where the respondents seemed to endorse the correct answer.

#### 3.2.2. Sources of Knowledge and Beliefs

A central question in this study is about how individuals learn about parenting and child development. Participants were asked where they learned what it means to be a parent (see Table 5). They were able to select one or more responses including “From a class”, “From books”, “From television or movies”, “From watching my own parents”, or “Other”. By far, students report learning about what it means to be a parent from their own parents (48% of respondents included this as one of their responses). Only 13% of responses included learning about parenting from a class, less than those who report learning from television or movies (16%). Another open-ended question asks, “Imagine you are a parent of a four-year-old. You want to make sure she is ready to start kindergarten when she is five. Who do you ask for advice?” This question, which is both more concrete (asking the respondent to imagine him- or herself in a scenario) and open-ended (respondents could write in any answer they chose) than the previous question, yielded similar results: The most common response was they would ask their own parent(s) (570 participants included this in their response). 

Only fifteen respondents reported that they would not ask anyone or would rely on their own judgement rather than asking for advice about their child’s readiness for kindergarten. High school students in the sample do not have much confidence that they will “just know what to do” when they become a parent (only 9.13% “Strongly Agree” and 19.94% “Agree” that they will know what to do).

#### 3.2.3. Gender

Compared to girls, boys are less likely to believe that holding a growth mindset was important (*d* = −0.22, *t*(966) = −3.48, *p* < 0.001), are less likely to believe in the importance of the caregiver’s role in promoting oral language development (*d* = −0.28, *t*(919) = −4.19, *p* < 0.001), and are less likely to believe in the importance of socio-emotional development (*d* = −0.28, *t*(899) = −4.16, *p* < 0.001). There did not appear to be any significant associations between a belief in the role that parents and caregivers play in early learning, the importance of active learning for children, and what it takes to be a parent. Girls’ and boys’ responses to questions about their knowledge of child development were similar (see Table 6). Boys were more likely to answer incorrectly and answer “Don’t know” (except for whether 4 years old was too young to do many things for themselves) compared with girls, although some of these differences were not statistically significant. For example, when asked whether it is true or false that “Children who are one-year-old should be able to stay away from things that could harm them” (correct answer = false), 50% of girls answered correctly compared with only 39% of boys. 

#### 3.2.4. Socio-Economic Status

We used two measures to estimate high school students’ socio-economic status: One at the individual level (maternal educational attainment) and one at the community level (median income for their zip code). Respondents were asked whether their mother did not finish high school (16%), received a high school diploma (33%), received an associate degree (22%), received a bachelor’s degree (17%), received a master’s degree (8%), or received a professional or doctoral degree (5%). We fit a series of models regressing scale scores from the various subsection of the APKAS on maternal education, which we treated as categorical. The only model in which education was a significant predictor was one regressing beliefs related to children’s active roles in their own learning on maternal education (F = 7.73, *p* = 0.006). In this model, we found that, in general, children whose mothers had higher levels of education tended to have higher scores on the APKAS subsection. We only found a very small but significant association with beliefs about the importance of active learning, where respondents with the highest mother’s education level were more likely to believe that active learning is important (R^2^ = 0.01). 

In addition to using maternal education to estimate socio-economic status, we also used a community measure to attempt to understand the relative effects of the broader culture on beliefs and knowledge: We used the median income for participants’ zip codes using data from the 2016 American Community Survey from the US Census Bureau. While this is a very blunt measure of SES, prior research suggests that cultural models of child-rearing, beyond family-specific models, may be an important influence on adolescents’ beliefs [2]. We used the ACS census data in a variety of ways to determine if there were any associations between median income and beliefs and knowledge. First, we assigned respondents to six median income categories and we looked for associations between attitudes and knowledge and these income levels: $0–$24,999; $25,000–$49,999; $50,000–$74,999; $75,000–$99,999; $100,000–$199,999; and $200,000 and higher (however, no respondents reported living in zip codes in the top median income category). As with participants’ maternal education, using linear regression we found that there are significant differences associated with attitudes about the importance of children playing active roles in their learning and different median income levels, where the higher the income category, the more likely the respondent is to believe that active learning is important for children (R^2^ = 0.02) (see Table 7). Unlike maternal education, there were also associations between the median income category and beliefs about the importance of empathy and socioemotional learning. Once again, respondents living in zip codes with higher income categories tended to believe that empathy and socio-emotional learning are more important (R^2^ = 0.01). However, there appears to be a negative relationship between income category and beliefs about the importance of the parent role, where the higher the income category, the less likely the respondent is to believe that caregivers should play an important role in early learning (R^2^ = 0.01).

We also tested associations using the log of income and found similar results. 

#### 3.2.5. Childcare Experience

Respondents reported how many times they had cared for a child younger than themselves in the past year (0 times, 1–4 times, 5–10 times, and more than 11 times). As shown in Table 8, high schoolers who had more childcare experience were more likely to hold beliefs consistent with positive outcomes for young children and their learning in all attitude categories and they were more likely to have more knowledge of child development compared with those with no experience or less experience.

There were also consistent significant differences between those who had no prior childcare experience and those who had even a moderate amount (5–10 times in the past year) for most of the belief categories (see Table 9). 

### 3.3. The Relationship between Knowledge of Child Development, Experience, Demographic Characteristics, and Parental Beliefs

**Figure 1:**
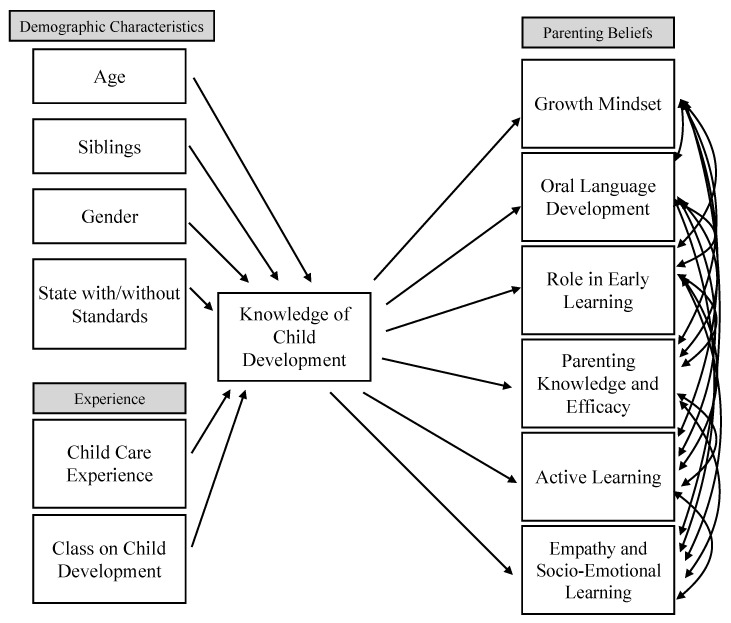
Proposed conceptual model of the relationships between demographic characteristics, experience, knowledge of child development, and parenting beliefs.

Our final goal was to use structural equation modeling to test our model of how beliefs about parenting and child development are shaped. We proposed a conceptual model where a combination of demographic characteristics and experiences would have an impact on beliefs, completely mediated by the individual’s knowledge of child development (see Figure 1). We tested the model for each belief category individually: The importance of children’s active learning, the importance of holding and promoting empathy and socio-emotional learning, the importance of holding and promoting a growth mindset, the importance of promoting oral language development, the importance of parenting knowledge and efficacy, and beliefs about the role that caregivers should play in early learning. The model also includes four demographic characteristics (age, gender, sibling status, and residing in a state with standards related to parenting and child development) and two experiences (childcare experience and having taken a class on child development). While in the previous analyses we treated knowledge of child development as an outcome variable, in this model, it is a mediator since prior research has shown that parental beliefs (and behaviors) are impacted by their knowledge of child development [13].

The fitted models shown in Table 10 indicate that having siblings, being female, and having prior childcare experience were often significant direct predictors of certain beliefs. More importantly, knowledge of child development was consistently a significant predictor across all belief categories. Having more knowledge of child development and more childcare experience are associated with beliefs about the importance of supporting and promoting active learning in young children, controlling for all of the other variables. However, having a younger sibling, controlling for everything else, is associated with less knowledge of child development. However, these associations are fairly weak.

More variables were associated with beliefs about the importance of empathy and socio-emotional learning, holding a growth mindset, and promoting oral language development than other belief categories. As with active learning, having a younger sibling had a small negative association. On the other hand, having more knowledge of child development, more childcare experience, being female, and taking a class on parenting or child development are associated with significant positive beliefs on empathy, growth, and oral language beliefs, controlling for all of the other variables. Finally, only having more knowledge of child development and more childcare experience are associated with positive beliefs about the importance of parenting knowledge and efficacy and the caregiver’s role in promoting early learning, controlling for all of the other variables.

## 4. Discussion

The goal of this study was to determine what a sample of American high school students knows and believes about several aspects of parenting and child development, particularly those aspects of parenting and child development that are believed to promote early learning, and the influences of those beliefs. We found that, in general, high school students are not very knowledgeable about the specifics of child development, based on a limited number of questions on typically agreed-upon child development milestones and trends. However, they do tend to believe that parents should play an important and proactive role in promoting early learning including oral language development, the development of socio-emotional skills, empathy, and a growth mindset, and that parenting requires some specialized knowledge and skills. Yet, many high school students do not feel very strongly about these beliefs, and a large proportion feels neutral about them. Furthermore, high school students report that, by far, they have learned the most about parenting from watching their own parents, and from television and movies next. This is especially troubling since it is likely that media depictions of the realities of parenthood and of the best strategies for promoting healthy development and academic success are lacking. At the same time, this phenomenon may also present an exciting opportunity to use the media to influence perceptions of parenting by promoting positive portrayals of model parenting.

We also tested a model to understand the different sources of information and factors that influence adolescents’ beliefs about parenting and child development. Using McGillicuddy-DeLisi and Subramanian’s theory of how children develop knowledge and beliefs as a conceptual framework, we constructed a new model where demographic characteristics (elements of their own culture) and experiences (their own childhood and exchanges of ideas through classes or other opportunities to learn about children and parenting) are mediated through their level of knowledge of child development to affect beliefs. Our results show that the most salient factors in predicting beliefs are knowledge of child development and prior childcare experience, with the gender of the individual and their sibling status also making some difference in some of the belief categories. From an intervention standpoint, however, gender and sibling status are not viable options for impacting beliefs but providing opportunities for gaining experience caring for young children and learning about child development could be promising. Our testing of this model suggests that it is a plausible interpretation of mechanisms through which beliefs are formed, but it is only a small piece of the picture of the overall influences on children’s understanding of what it means to be a parent and their role in processes of child development (as evidenced by the small effect sizes). However, there are some specific pathways to beliefs that are interesting and warrant further investigation.

One interesting finding involves the possible impact of sustained or intensive experience with young children. While higher levels of previous childcare experience (which may be in the form of babysitting) were associated with more desirable beliefs, some of the more hands-on/real experiences (being a teen parent, having a sibling in the home) seemed to *decrease* desirable beliefs. This echoes prior research that shows that hands-on experience with young children is useful for ensuring that instruction related to parenting and child development “sticks” [14,15]. It is also likely that those with more positive views and knowledge of child development are more likely to engage in childcare experiences.

We suggest that there may be an important difference between intensive and/or sustained experience with children and more occasional or casual child-rearing experiences (such as would be the case with babysitting or nannying). This paradoxical relationship between experience and beliefs may be the result of the difference between respondents answering under idealized conditions (for example, reading each item as “Parents should ideally…”) versus truly understanding the reality of looking after young children, which can be overwhelming, especially to a young person. This may not be so surprising since prior research has indicated that there may be an inverse relationship between how much adolescents know about parenting and child development and their feelings of competence or readiness to become parents [16]. The fact that being a teen parent is associated with less desirable beliefs also may suggest that the beliefs that individuals, including adolescents who are still maturing, hold about parenting are not stable [17], and that they may change when individuals actually become parents, which would have important implications for any attempt to affect parenting beliefs by targeting adolescents. Alternatively, it may be the case that those adolescents who become teen parents already generally hold negative beliefs about what it means to parent, potentially based on how they were parented, their sociocultural context, or some other factor. In addition, it could be argued that the “desirable” beliefs could be considered ambitious or more expansive than a more limited view of the role of parents (to feed, clothe, and love children only, but not necessarily serve as a teacher or person responsible for achieving academic learning outcomes for young children). It might be the case that teen parents (and other respondents) subscribe to a more limited view of parenting based on their own capacities as a teen parent or what they have observed from their own parents’ parenting style. Prior research has suggested that the differences between more limited versus more comprehensive parenting role conceptions may be class-based and/or cultural [3,18].

Additionally, substantial prior research has shown that there are socio-economic-based differences in child-rearing practices and attitudes, but most of that research has shown how low-income families differ from middle-class and higher-income families. This study, on the other hand, found few differences based on the respondent’s mothers’ education level but found some differences between students from middle-class communities compared to communities at both higher and lower income levels (using respondents’ zip codes’ median income). If there is a middle-class effect, then it could be because, compared with the most wealthy and the least wealthy, middle-class parents have the most time for their children and can model desirable parenting behaviors and attitudes. Since most high school students reported learning about parenting and child development from watching their own parents, high school students who have had positive parenting role models might tend to hold more desirable beliefs.

This study has several implications for policy, programming, and for future research. First, the APKAS is an instrument that can be used in future research to help us understand what adolescents know and believe about those aspects of parenting related to early learning, and how they come to know these things. Second, by identifying seven dimensions of parenting related to early learning through a review of the literature, this study provides policymakers and educators with seven recommendations that may be useful to promote in adolescents:

1. Children should play an active role in their own learning.
2. Parents should be empathetic toward their children and promote socio-emotional learning in them as well.
3. Caregivers should praise their children’s efforts, in addition to their intelligence.
4. Caregivers should actively promote oral language development by talking with and listening to their children, even before they can speak properly.
5. Parenting skills can be learned, and parenting is difficult and requires patience.
6. Caregivers play a key role in children’s early learning.
7. Knowing more about child development can have a positive effect on developing the beliefs listed above.

While this study supports the idea that providing high school students with more information about children’s development and basic developmental milestones can lead them to holding desirable beliefs about parenting and the parent’s role in early learning, in general, it is less clear how to provide that information. More knowledge of child development is associated with more desirable beliefs, as shown in the conceptual model we proposed of the mediating role of knowledge on experience, characteristics, and beliefs.

Therefore, a class that uses the results of this study to build upon students’ already fairly strong beliefs about the important role that parents and other adults can play in children’s early learning could be useful. In addition, the findings suggest that while some students hold desirable beliefs, they may not have clear ideas about how, practically speaking, adults can promote early literacy, numeracy, and socio-emotional development in young children. A class could also be useful by providing high school students with reliable sources of information for learning about parenting and child development—in addition to their own parents and the media, both of which may or may offer positive role models—and ideas for who to turn to if they have questions about the children they look after or who they can ask when and if they are parents someday.

It is our hope that this research provides the first glimpse into a broad sample of American adolescents’ views on parenting and child development, including what they know, how they have come to learn about parenting and child development, and their beliefs. If parental beliefs reflect cultural values [19], this study shows that many high schoolers hold parenting beliefs that would lead to their children’s early learning success but need more specifics about the process of child development and the role of adults in that process, and perhaps we should be more intentional, as a society, about the parenting values that we hope that our future parents develop.

## 5. Limitations

One of the key limitations of this study is the challenge of interpreting results given the tendency for individuals, especially those with low levels of education (which could include high school students), to agree to opinion questions. However, after a careful review of both the drawbacks of agree–disagree question forms and alternative question forms, we determined that it is well-suited for this study. First, agree–disagree questions are useful for determining respondents’ attitudes and opinions, a primary goal of this research. Second, as agree–disagree questions are among the most commonly used, it is likely that adolescents will have some familiarity with the question format. Third, alternative question formats tend to be unwieldy and difficult to interpret by participants, and therefore may raise other issues that would undermine confidence in the results. 

A second limitation of this study is the generalizability of the study. Although we sought to recruit a sample that reflects current US demographic trends, it may not be possible to make claims about the population of US high school students based on the results from the Qualtrics sample. It is very likely that the sample we recruited differed from the population in terms of both observable and unobservable characteristics. However, despite these limitations, this study still contributes to what little is currently known about high school students’ beliefs and knowledge about parenting and child development. To our knowledge, no previous studies have included such a large and diverse sample, nor have they focused on students that are not teen parents or considered at risk of teen parenthood.

Third, our index of knowledge of child development would benefit from further revision. There is low internal consistency (α = 0.31) among the items on this index. The items were chosen to particularly target knowledge related to what young children are capable of at particular ages and their ability to learn. It is likely that the adolescents in the sample have very little knowledge of these topics since their answers are roughly as good as (or worse) than if they guessed. While we ultimately decided to retain this index for this study because of the centrality of the knowledge of what children are capable of and how they learn the theory we are building, further refinement of the index may be warranted in the next version of the APKAS.

Finally, we expected larger associations between socio-economic status and beliefs than we ultimately found. While these modest relationships with income are somewhat surprising given the prior research on the importance of socio-economic status on knowledge of child development, it is important to note that these findings may not accurately reflect the socio-economic status of the respondents, since the median income of the respondents’ zip codes is a fairly blunt measure. Instead, these findings might represent, to some extent, the socio-cultural context in which the respondents are living, which is believed to have an impact on parenting beliefs [1].

## Figures and Tables

**Table 1:** Participants’ demographic information.

	Quota (%)	*n*	%
Total		1044	100
Grade			
9th grade	15–25	84	8.33
10th grade	15–25	187	18.55
11th grade	25–35	369	36.61
12th grade	25–35	368	36.51
Age *			
13		17	1.63
14		45	4.31
15		98	9.39
16		381	36.49
17		279	26.72
18		207	19.83
19		17	1.63
Gender			
Female	56	581	55.65
Male	44	463	44.35
Race			
African-American	15–20	150	14.37
White	70–80	702	67.24
Asian or Pacific Islander	5–10	89	8.52
More than one race *		34	3.26
Other *		70	6.70
Hispanic			
Yes	25	263	25.19
No	75	781	74.81
Maternal education			
Did not graduate from high school	Up to 30	156	15.48
High school diploma	Up to 30	316	31.35
Associate degree	20	222	22.02
Bachelor’s degree	15	173	17.16
Master’s degree	5	92	9.13
Doctoral or professional degree		49	4.86
Geographic Region			
Midwest (IA, IL, IN, KS, MI, MN, MO, ND, NE, OH, SD, or WI)	26	241	23.08
Northeast (CT, MA, ME, NH, NJ, NY, PA, RI or VT)	18	171	16.38
Pacific and West (AK, AZ, CA, CO, HI, ID, MT, NM, NV, OR, UT, WA, or WY)	14	200	19.16
South (AL, AR, DC, DE, FL, GA, KY, LA, MD, MS, NC, OK, SC, TN, TX, VA, or WV)	42	432	41.38

* Note: No quotas for these categories.

**Table 2:** Percentage of participants’ answers regarding their beliefs within each category.

	Don’t Know	Strongly Disagree	Disagree	Neutral	Agree	Strongly Agree
Active learning						
Learnbytrying	3.08	1.19	1.29	11.61	45.34	37.50
Curious	2.19	1.09	2.19	12.20	36.61	45.73
Dothemselves	2.18	1.79	2.98	25.40	40.28	27.38
Explore	2.68	1.39	3.27	19.35	43.85	29.46
Vignette	3.57	6.65	17.36	25.60	35.02	11.81
Learninterested	2.08	1.09	0.89	8.93	33.23	53.80
Empathetic awareness & socio-emotional learning						
Vignette	2.98	1.19	2.58	10.02	43.65	39.58
Maketrouble	4.07	2.38	2.78	12.70	33.53	44.54
Parentssensitive	6.75	15.48	25.99	30.95	15.87	4.96
Playnicely	1.98	0.99	2.18	7.54	31.25	56.05
Feelingsothers	2.58	1.29	2.38	9.03	36.81	47.92
Responddifferently	6.25	1.59	3.08	14.19	46.43	28.47
Otherperspectives	2.09	1.29	1.98	10.81	39.58	44.25
Growth mindset						
Praisegood	3.17	1.59	2.18	11.31	34.52	47.22
Parentsmatter	2.88	1.49	2.38	9.23	32.94	51.09
Alwaysencourage	1.69	1.09	2.08	6.65	27.38	61.11
Learnparent	2.28	1.09	1.88	10.62	36.31	47.82
Vignette	4.07	3.97	7.34	14.68	36.81	33.13
Oral language						
Allkindsofwords	4.56	2.48	8.04	21.92	36.61	26.39
Readoften	2.38	1.59	1.79	15.58	41.17	37.50
Fullexplanation	3.17	1.49	4.46	35.42	33.63	21.83
Talktobabies	3.90	1.79	1.29	10.71	34.72	47.62
Twentyquestions	6.55	1.19	5.16	22.82	40.77	23.51
Childrentalk	2.38	1.49	1.49	8.23	40.48	45.93
Parenting knowledge & efficacy						
Controltemper	2.18	1.49	2.28	11.41	35.22	47.42
Patient	2.08	0.79	1.19	8.04	31.94	55.95
Nurtureself	13.69	1.49	5.36	35.42	28.47	15.58
Goodjob	9.14	1.23	2.46	17.76	34.50	34.91
Hard	8.43	1.49	4.37	19.15	29.96	36.61
Knowalot	2.98	1.59	5.85	20.93	39.29	29.37
Role in early learning						
Highexpectations	3.47	3.77	13.79	36.61	26.19	16.17
Play	3.77	1.09	1.95	10.22	41.96	41.07
Beforeschool	2.58	1.19	2.48	16.17	41.17	36.41
Teachalphabet	4.56	1.49	5.16	29.07	34.33	25.40
Everydaymath	3.08	1.19	2.58	17.26	46.43	29.27

Note: Negatively worded items have been recoded to match the scale of positively worded items.

**Table 3:** Correlations between belief measure scale scores.

Variable	Active	Empathy	Growth	Oral	Parenting
Active					
Empathy	0.601 **				
Growth	0.576 **	0.651 **			
Oral	0.604 **	0.658 **	0.577 **		
Parenting	0.561 **	0.606 **	0.588 **	0.629 **	
Role	0.422 **	0.466 **	0.463 **	0.567 **	0.592 **

** *p* < 0.01.

**Table 4:** Percentage of participants’ responses to questions about their knowledge of child development.

	Don’t Know	True	False	Preferred Answer
Children age four and under are too young to do many things for themselves.	12.70	51.39	35.91	F
Children as young as two months old can get bored.	33.73	45.63	20.63	T
A one-year-old knows right from wrong.	17.36	13.99	68.65	F
Children who are one-year-old should be able to stay away from things that could harm them.	13.10	42.26	44.64	F
Babies usually say their first real word at six months.	44.44	31.35	24.21	F
When 18-month-old children point at things, they are trying to show others something that they find interesting.	18.35	74.60	7.04	T

**Table 5:** Responses to “Imagine you are a parent of a four-year-old. You want to make sure she is ready to start kindergarten when she is five. Who do you ask for advice?”.

	*n*	%
Own parents	570	44.57
Teacher, counselor, or school official	267	20.88
Other parents or friends with children	178	13.92
Other family members (grandparents, siblings, or others)	71	5.55
Internet/Google/books	35	2.74
Pediatrician or doctor	28	2.19
No one/self	15	1.17
Spouse	14	1.09
Child	6	0.47
Don’t know	21	1.54
Other	74	5.79

Note: “Other” responses include gibberish and off-topic answers.

**Table 6:** Percentage of participants’ responses to questions about their knowledge of child development by gender.

	Female			Male			Preferred Answer			
	Don’t Know	True	False	Don’t Know	True	False		χ^2^	df	*p*
Tooyoung	13.08	47.29	39.63	12.61	55.75	31.64	F	6.57	1	0.01
Getbored	32.52	45.35	22.24	36.06	45.35	18.58	T	0.03	1	0.87
Knowright	17.20	13.46	69.35	18.14	14.82	67.04	F	0.99	1	0.32
Stayaway	13.27	37.20	49.53	13.27	48.23	38.50	F	13.88	1	0.00
Firstword	41.12	32.15	26.73	48.23	30.09	21.68	F	3.60	1	0.06
Pointing	16.82	76.07	7.10	20.35	72.79	6.86	T	2.17	1	0.14

**Table 7:** Associations between respondents’ zip code’s median income category and APKAS responses by category.

	Active	Empathy	Growth	Oral	Parenting	Role	KOFCD
(Intercept)	3.80 ***	3.91 ***	4.24 ***	4.09 ***	4.09 ***	4.11 ***	0.47 ***
	(0.06)	(0.06)	(0.06)	(0.07)	(0.07)	(0.06)	(0.02)
Median income	0.09 ***	0.05 *	0.02	−0.00	−0.00	−0.05 *	0.01
	(0.02)	(0.02)	(0.02)	(0.02)	(0.02)	(0.02)	(0.01)
R^2^	0.02	0.01	0.00	0.00	0.00	0.01	0.00
Adj. R^2^	0.02	0.01	−0.00	−0.00	−0.00	0.01	0.00
Num. obs.	873	814	879	836	698	844	943
RMSE	0.55	0.53	0.58	0.60	0.57	0.58	0.22

*** *p* < 0.001, * *p* < 0.05.

**Table 8:** Associations between prior childcare experience and APKAS responses by category.

	Active	Empathy	Growth	Oral	Parenting	Role	KOFCD
(Intercept)	3.97 ***	3.94 ***	4.15 ***	3.98 ***	3.98 ***	3.83 ***	0.44 ***
	(0.03)	(0.03)	(0.04)	(0.04)	(0.04)	(0.04)	(0.01)
Childcare experience	0.05 **	0.05 ***	0.08 ***	0.05 **	0.05 **	0.06 ***	0.03 ***
	(0.02)	(0.02)	(0.02)	(0.02)	(0.02)	(0.02)	(0.01)
R^2^	0.01	0.01	0.02	0.01	0.01	0.01	0.02
Adj. R^2^	0.01	0.01	0.02	0.01	0.01	0.01	0.02
Num. obs.	965	901	968	921	770	936	1044
RMSE	0.55	0.52	0.58	0.60	0.56	0.58	0.22

*** *p* < 0.001, ** *p* < 0.01.

**Table 9:** Association between respondents with no childcare experience versus those with a moderate amount of childcare experience and the APKAS responses by category.

	Active	Empathy	Growth	Oral	Parenting	Role	KOFCD
(Intercept)	4.04 ***	4.03 ***	4.31 ***	4.07 ***	4.09 ***	3.94 ***	0.49 ***
	(0.03)	(0.03)	(0.04)	(0.04)	(0.04)	(0.04)	(0.01)
No childcare experience versus those with 5–10 times in the past year	−0.21 *	−0.16	−0.32 **	−0.22 *	−0.24 *	−0.17	−0.14 ***
	(0.10)	(0.10)	(0.11)	(0.11)	(0.11)	(0.10)	(0.04)
R^2^	0.02	0.01	0.03	0.01	0.02	0.01	0.04
Adj. R^2^	0.01	0.01	0.03	0.01	0.01	0.01	0.04
Num. obs.	283	263	288	277	229	283	316
RMSE	0.53	0.52	0.61	0.59	0.54	0.57	0.23

*** *p* < 0.001, ** *p* < 0.01, * *p* < 0.05.

**Table 10:** Structural equation model of beliefs, demographic characteristics, and previous experience and knowledge.

	Active	Empathy	Growth	Oral	Parenting	Role
KOFCD	2.63 ***	2.59 ***	3.22 ***	4.20 ***	3.13 ***	1.79 ***
	(0.64)	(0.76)	(0.83)	(1.12)	(0.94)	(0.55)
	**KOFCD**	**KOFCD**	**KOFCD**	**KOFCD**	**KOFCD**	**KOFCD**
Age	0.009	0.006	0.01	0.004	0.01 *	0.01 *
	(0.005)	(0.004)	(0.004)	(0.004)	(0.004)	(0.004)
Siblings	−0.03 **	−0.03 *	−0.03 *	−0.03 **	−0.02	−0.02 *
	(0.01)	(0.01)	(0.01)	(0.01)	(0.01)	(0.01)
Female	0.02	0.03 **	0.03 *	0.03 **	0.02 *	0.01
	(0.01)	(0.01)	(0.01)	(0.01)	(0.01)	(0.01)
State with standards	0.02	0.01	0.01	0.02	0.02	0.02
	(0.01)	(0.01)	(0.01)	(0.01)	(0.01)	(0.01)
Childcare experience	0.02 **	0.02 **	0.02 **	0.02 **	0.02 **	0.02 **
	(0.007)	(0.01)	(0.01)	(0.01)	(0.01)	(0.01)
Class	0.03 *	0.03 *	0.02 *	0.02	0.03 *	0.02 *
	(0.01)	(0.01)	(0.01)	(0.01)	(0.01)	(0.01)
Num. obs.	1015	1015	1015	1015	1015	1015
χ^2^	253.71	289.64	262.73	309.22	280.99	208.93
df	119	136	103	119	119	103
*p*-value	0.00	0.00	0.00	0.00	0.00	0.00
RMSEA	0.03	0.03	0.04	0.04	0.04	0.03
CFI	0.91	0.92	0.89	0.89	0.84	0.89
SRMR	0.04	0.04	0.04	0.04	0.04	0.04

*** *p* < 0.001, ** *p* < 0.01, * *p* < 0.05. Note: We used analytical standard errors in testing for mediation.

## Data Availability

The data that support the findings of this study are available from the corresponding author upon reasonable request.

## Supplementary Materials

The following supporting information can be downloaded at: https://www.mdpi.com/article/10.3390/children10010025/s1. Supplement: Developing and Validating the APKAS. Table S1. Demographic profile of pilot sample (n = 102). Table S2. Summary statistics for confirmatory factor analysis of ordinal measures during pilot. Table S3. Summary statistics for validation of measures. Table S4. Measurement invariance across demographic categories. Table S5. Correlations between belief measure scale-scores.
