# Supplementary material for: American High School Students’ Knowledge and Beliefs about Parenting and Early Childhood Development
Source: Children (Basel). 2022 Dec 23;10(1):25. doi: 10.3390/children10010025 (PMC9856310; doi:10.3390/children10010025)
Supplement: Supplementary file 1 [file children-10-00025-s001.zip › children-1967060-supplementary.pdf]

Supplement: Developing and Validating the APKAS

1. Introduction

To make progress toward understanding what American high school students know and believe about parenting and child development (RQ1), to understand how knowledge and beliefs differ across groups (RQ2), and to understand the relationship between knowledge of child development, demographic characteristics, child care experience, and beliefs about parenting and child development (RQ3), we designed, validated, and administered a questionnaire, the Adolescent Parenting Knowledge and Attitudes Survey to a sample of American high school students (N=1,044). To develop the questionnaire, we first reviewed existing studies of adolescent parenting knowledge and attitudes, including the methods they used, as well as existing questionnaires, to determine whether an instrument already existed to assess adolescents' beliefs and knowledge about the role of parenting in early learning. This review of existing questionnaires also helped to understand the gap this study addresses and helped to clarify what should be included and excluded from the new questionnaire used in this study. Next, we reviewed state standards to understand what state education departments currently prioritize in terms of knowledge of parenting and child development. In the third step, we used information from the previous two steps as well as key research on promoting healthy development and early learning to define the constructs and categories for inclusion on the new questionnaire. Finally, in the fourth step, we describe the process through which we sought to determine whether the new questionnaire could be considered valid and to establish the reliability of the various scales for understanding adolescent knowledge and beliefs related to child development and parenting.

2. Review of existing literature and questionnaires

The process of creating the questionnaire first involved a review of literature on the beliefs related to early learning and child development that should be included in the survey as well as existing studies and questionnaires related to parenting knowledge and attitudes and knowledge of child development for both adolescents and adults. As noted above, a review of the literature indicates that very few studies have been conducted on the topics of interest with adolescents, and fewer have used surveys to understand adolescent attitudes and knowledge.

To create a sample of existing questionnaires for review, we conducted a search in ProQuest's PsychTests databased to limit results to instruments. We used various permutations of the following search terms to find results:

- Parent*.
- Child development.
- Adolescent*.
- High school*.
- Questionnaire.

Through this search and by adding any other surveys we came across during our reading of the relevant literature, we were able to find twenty-eight non-unique measurement instruments. We reviewed all of the questionnaires for their content, their audience, and their structure (e.g., multiple choice questions; What kind of scale? How many items?). We then noted all of the items that seemed relevant to our purpose of learning about what high schoolers know and think about aspects of parenting and child development related to early learning.

From this initial process, we compiled a series of questions/items, which formed the first draft of the questionnaire. Although many of the questionnaires we reviewed sought to understand behaviors and attitudes related to child abuse and child abuse prevention, since child abuse prevention is not a primary goal of this research, many questions/items were not relevant for inclusion on the APKAS. We then reviewed

the compiled list and eliminated duplicates, removed irrelevant questions, and reworded questions. For example, we adapted the following question from the Adult Adolescent Parenting Inventory-Version 2 (AAPI-2):

Original from AAPI-2: *Praising children is a good way to build their self-esteem.*
Revised item for APKAS: *Praising children's hard work is a good way to build their self-esteem.*

We added "hard work" to align more closely with research on the importance of effort in the theory of growth mindset.

3. Determining the constructs and categories of information to be used in the survey

Next, we categorized the questions to determine whether there were natural groupings and constructs that emerged from this emic process that we could compare with the topics that we had previously identified as important through the review of the literature. The items that we selected fell into the following topic categories:

- Biographical information.
- Growth mindset.
- Role of parents.
- What can children learn/do.
- Empathetic awareness.
- Self-regulation.
- Early literacy and math.
- Parenting efficacy.

These were among the topics that we had identified at the outset of this research project would be useful to learn about. None of the questionnaires we reviewed included questions on parenting efficacy that we felt were worth including in the APKAS since research has shown that parents' sense of competence is associated with child outcomes and parenting behaviors, which, in turn, affect child outcomes.

Initially the questions were grouped into four categories: Questions related to growth mindsets, the role of parents (including how they see their role in promoting early learning and whether they feel a sense of efficacy), expectations for children (including what kinds of literacy and numeracy skills children can be expected to acquire at young ages), and questions related to empathy and socio-emotional awareness. These four groupings represent key areas of knowledge necessary for caregivers to support young children's learning. These were later revised based on the results of the pilot (details below). In this way, we operationalized "parenting beliefs" to include beliefs about the role parents should play in early learning, the importance of oral language development, beliefs about promoting active learning for children, the importance of empathy for children and parents, beliefs about parenting knowledge and self-efficacy, and holding a growth mindset.

The final version of the APKAS questionnaire includes 78 items relating to the following seven categories known to relate to parenting practices and demographic information: Active learning, empathy, growth mindset, oral language development, parenting, role in early learning, knowledge of child development, biographic information, sources that inform respondents' knowledge and attitudes, and final open questions (see page 4 of article for more information about the content of the APKAS). The categories of items were chosen for inclusion by the process outlined above, and also because of evidence of their importance for children's healthy development and early academic. The items were presented in blocks, with one block for each category of item (demographic information, active learning, empathy and socio-emotional learning, etc.). In addition to the first block with biographical questions and the final block, which

included questions about sources of parenting knowledge and the final open-ended questions, the blocks were randomized at two levels: Items within each block were presented in random order, and the blocks themselves were presented in random order.

4. Validation of the APKAS

The APKAS questionnaire was validated the through the following process: (1) It was reviewed by parenting and child development experts to ensure that the content of the questionnaire was appropriate and accurate; (2) the content of the questionnaire was cross-referenced with state standards related to parenting and child development; and (3) the internal validity of the constructs was established through confirmatory factor analysis (CFA) with pilot data. Prior to conducting the pilot, we first surveyed fifty individuals for a "preliminary pilot" to test the survey's flow and ensure that all proper conditions were in place to automatically end the survey for ineligible respondents (such as adults or individuals outside the United States), etc. In addition, we tested the internal consistency of each measure, which was confirmed using Cronbach's alpha.

4.1 Sharing and Revision/Expert Review

The APKAS was shared with two high schoolers and two "experts," a pediatrician and a researcher. The high school students were selected by convenience, and each completed the questionnaire and discussed any questions that seemed confusing, ambiguous, irrelevant, or difficult to answer. Their responses were not used in any other way. The experts were chosen because they presented two different kinds parenting expertise. Each of the experts were asked to review the questionnaire and respond to the following questions:

- Is the content appropriate for a questionnaire that seeks to understand adolescents' parenting attitudes and knowledge of child development?
- Is there anything missing?
- Do you think that the results of the questionnaire will uncover differences in adolescents' parenting attitudes and knowledge of child development?
- Do you have any additional thoughts or comments?

A few examples of changes suggested by the experts and high school students and the action taken in response are as follows:

- Consider adding a question about parents' primary language as a proxy for culture, since we know this matters for parenting and childrearing attitudes (pediatrician): Ultimately, language was not included in the questionnaire (beyond asking if the respondent was from a Hispanic background) since the APKAS already included many demographic questions.
- Changing the word "development" to "learning" in Q6.5 (pediatrician): This change was made.
- High school student was confused by the item: "Parents should not be too affectionate because it might make the child weak." Found it difficult to answer. The student's confusion caused us to question the value of this question and it was ultimately removed from the questionnaire.

4.2 Preliminary pilot

The draft *Adolescent Parenting Knowledge and Attitudes Survey* was administered to 50 individuals via Qualtrics and recruited via Amazon's Mechanical Turk service. Mechanical Turk is service where individuals can volunteer to complete a task, usually for a small fee (Respondents who qualified and who completed the survey received between $1 and $1.50.

). Out of 48 usable responses (Two respondents were excluded because they failed to confirm that they were in fact a high school student and not the parent completing the questionnaire.), the respondents came from 23 states, and were fairly evenly spread across the expected age and grade ranges. More boys completed the survey than girls (29 versus 19).

The preliminary testing was useful for refining the measures. We conducted confirmatory factor analysis with each of the categories measured by the APKAS to determine fit. This led to significant reorganization to the instrument (but not substantive changes to the items).

4.3 Pilot

Following the preliminary pilot, we conducted a larger pilot with 102 respondents to further refine the measures. We conducted a series of tests to understand how the different items in each category related to one another and to determine whether they were measuring the same underlying construct. First, we explored correlations between the items to determine whether people answered similarly across all items in the category. If an individual item seemed not to correlate with any of the other items in the category, we considered whether that item should be eliminated or reworded. For example, the responses given by the pilot sample on one item related to the construct related to the respondents' belief in the importance of parents encouraging oral language development, "There is no point in talking to babies who can't talk yet," did not correlate well with other items within the same category. To rectify this, we reworded the question so that it was positively worded: "Talking to babies—even if they can't talk yet—is important."

Table S1. Demographic profile of pilot sample (n=102).

	Category	Frequency	Percentage
Gender	Female	51	55%
	Male	63	45%
Grade	9th	30	26%
	10th	25	22%
	11th	36	32%
	12th	23	20%
Race/Ethnicity	Caucasian	83	73%
	African-American	12	11%
	Hispanic/Latino	8	7%
	Asian/Pacific Islander	2	2%
	Other or More than One Race/Ethnicity	9	8%

We next conducted confirmatory factor analysis (CFA) with each category to make revisions to individual items, which resulted in the elimination or rewriting of additional items that, according to the analytic results, did not accurately measure the same underlying construct as other items in the category. We also used the root mean square error of approximation (RMSEA<0.08) and comparative fit index (CFI≥ 0.90) to check for approximate model-to-data fit (see Table S2). Finally, we examined the modification indices to see the correlation between the items that could not be explained by the factor in order to determine whether any items should be eliminated or rewritten.

Table S2. Summary statistics for confirmatory factor analysis of ordinal measures during pilot.

Measure	RMSEA	CFI	χ^2	*P*
Active learning	0.158	0.992	7.315	0.136
Empathy and socio-emotional learning	0.000	1.000	5.580	0.781
Growth	0.000	1.000	1.400	0.992
Oral language	0.054	1.000	11.568	0.773

Parenting skills and efficacy	0.012	1.000	9.109	0.912
Role in early learning	0.000	1.000	5.580	0.781
Knowledge of child development	0.000	1.000	0.086	0.961

Note: Kline's thresholds for fit are as follows: χ^2 *p-value > 0.05; CFI ≥ 0.90; RMSEA < 0.08.*

All of these steps led to the rewriting of several items, the elimination of a few very poorly fitting items, the addition of a few items, and a better understanding of the relationship between each of the items and the underlying constructs we were trying to measure.

With the full sample of 1044 respondents, we repeated the tests from the pilot and examined the reliability of the measures using Cronbach's alpha (α). As seen in Table S3, the scales have high internal consistency. On the other hand, the α for the knowledge of child development items is low and could use further refinement in a second version of the measure. This index of child development knowledge particularly targets knowledge related to understanding what young children are capable of at particular ages and their ability to learn. It is likely that the adolescents in the sample have very little knowledge of these topics since their answers are roughly as good as (or worse) than if they guessed. While we ultimately decided to retain this index for this study because of the centrality of the knowledge of what children are capable of and how they learn to the theory we are building in this study, further refinement of the index may be warranted in a next version of the APKAS.

We also checked the CFA model for each construct, assuming the indicators are ordinal variables. We examined the models' chi-squared tests to test a null hypothesis of exact model-to-data fit since in a sample of 1044, there is the possibility of rejecting chi-squared even if the model fit is reasonably good. The root mean square error of approximation (RMSEA) and comparative fit index (CFI) for each of the measures also indicate excellent model-to-data fit. Following the CFA with each category, we conducted an omnibus CFA with all of the belief categories included. The low RMSEA (0.049) and CFI (0.893) just shy of Kline's threshold gave us confidence in the validity of questionnaire's measurement of parenting beliefs.

Table S3. Summary statistics for validation of measures.

Measure	Cronbach's α	RMSEA	CFI	χ^2	*P*
Active learning	0.74	0.000	1.000	2.768	0.973
Empathy and socio-emotional learning	0.74	0.013	1.000	16.236	0.299
Growth	0.72	0.042	0.999	13.563	0.019
Oral language	0.78	0.026	0.999	14.686	0.100
Parenting skills and efficacy	0.71	0.031	0.996	15.677	0.074
Role in early learning	0.69	0.036	0.997	11.173	0.048
Knowledge of child development*	0.31	0.045	0.962	27.822	0.001
Omnibus of belief measures		0.049	0.893	1274.29	0.000

Note: Kline's thresholds for fit are as follows: χ^2 *p-value > 0.05; CFI ≥ 0.90; RMSEA < 0.08. Cronbach's* α.
**Excludes "Don't Know" responses.*

Next, we looked at measurement invariance to ensure that the measures were functioning similarly for adolescents from each of the demographic categories that we included in our final structural equation model: Age, gender, sibling status, and residing in a state with standards related prior childcare experience and having taken a class on child development. We used three different metrics to measure for invariance, ΔCFI, ΔRMSEA, and ΔSRMR. Based on these three methods, we found that the measures worked equally well for all of the demographic categories (see Table S4). For gender, we found metric invariance based on all three metrics (ΔCFI<0.005; ΔRMSEA<0.01; ΔSRMR<0.03) and scalar invariance based on only two metrics

(ΔRMSEA<0.015; ΔSRMR<0.015). As far as we can tell, these measurements function similarly for boys and for girls, and therefore differences in results for gender are meaningful, as with each of the other demographic categories.

Table S4. Measurement invariance across demographic categories.

	Metric invariance			Scalar invariance		
Measure	ΔCFI	ΔRMSEA	ΔSRMR	ΔCFI	ΔRMSEA	ΔSRMR
Age	0.000	0.001	0.005	0.003	0.000	0.001
Gender	0.001	0.001	0.004	0.012	0.002	0.002
Sibling status	0.003	0.000	0.006	0.002	0.000	0.001
State with standards	0.001	0.001	0.004	0.002	0.000	0.001
Childcare experience	0.005	0.000	0.015	0.005	0.000	0.001
Taken class on child development	0.001	0.001	0.003	0.001	0.001	0.004

Note: Chen's thresholds for fit are as follows: ΔCFI < 0.006; ΔRMSEA < 0.01; ΔSRMR <0.03.

Finally, we looked at correlations between each of the scale scores of each construct to confirm that they all relate to one another as aspects of parenting beliefs and knowledge (see Table S4). In some cases, the correlations are weaker, such as the correlation between respondents' beliefs about the role that parents should play in early learning and beliefs about the importance of holding a growth mindset (r=0.463), the correlation between beliefs about role and the importance of active learning (r=0.422), and the correlation between the role and the importance of promoting empathy and socio-emotional skills in children (r=0.466). In all other cases, the correlations overall are moderately strong (r=0.50-0.60). This information, along with the research that suggests that these constructs are important elements of parenting beliefs, suggests that the instrument provides useful information about respondents' beliefs about parenting.

Table S5. Correlations between belief measure scale-scores.

Variable	Active	Empathy	Growth	Oral	Parenting
Active					
Empathy	0.601				
Growth	0.576	0.651			
Oral	0.604	0.658	0.577		
Parenting	0.561	0.606	0.588	0.629	
Role	0.422	0.466	0.463	0.567	0.592

5. Summary

The process of developing and validating the APKAS has given us strong reason to believe these scales measure single underlying constructs and that they are at least moderately reliable. Since there is at least a moderate degree of correlation between the constructs, we have confidence that the *Adolescent Parenting Knowledge and Attitudes Survey* is an accurate and reliable instrument for understanding aspects of American high school students' knowledge and beliefs about between parenting and child development.
